# Analysis of rhodopsin G protein-coupled receptor orthologs reveals semiochemical peptides for parasite (*Schistosoma mansoni*) and host (*Biomphalaria glabrata*) interplay

**DOI:** 10.1038/s41598-022-11996-x

**Published:** 2022-05-17

**Authors:** Phong Phan, Di Liang, Min Zhao, Russell C. Wyeth, Conor Fogarty, Mary G. Duke, Donald P. McManus, Tianfang Wang, Scott F. Cummins

**Affiliations:** 1 Centre for Bioinnovation, University of the Sunshine Coast, Maroochydore, QLD 4558 Australia; 2 School of Science, Technology and Engineering, University of the Sunshine Coast, Maroochydore, QLD 4558 Australia; 3 Department of Biology, St. Francis Xavier University, Antigonish, NS B2G2W5 Canada; 4 Molecular Parasitology Laboratory, QIMR Berghofer Medical Research Institute, Brisbane, QLD 4006 Australia

**Keywords:** Transcriptomics, Proteomics, Pathogenesis, Infection

## Abstract

Schistosomiasis is a medically significant disease caused by helminth parasites of the genus *Schistosoma*. The schistosome life cycle requires chemically mediated interactions with an intermediate (aquatic snail) and definitive (human) host. Blocking parasite development within the snail stage requires improved understanding of the interactions between the snail host and the *Schistosoma* water-borne free-living form (miracidium). Innovations in snail genomics and aquatic chemical communication provide an ideal opportunity to explore snail-parasite coevolution at the molecular level. Rhodopsin G protein-coupled receptors (GPCRs) are of particular interest in studying how trematode parasites navigate towards their snail hosts. The potential role of GPCRs in parasites makes them candidate targets for new antihelminthics that disrupt the intermediate host life-cycle stages, thus preventing subsequent human infections. A genomic-bioinformatic approach was used to identify GPCR orthologs between the snail *Biomphalaria glabrata* and miracidia of its obligate parasite *Schistosoma mansoni.* We show that 8 *S. mansoni* rhodopsin GPCRs expressed within the miracidial stage share overall amino acid similarity with 8 different *B. glabrata* rhodopsin GPCRs, particularly within transmembrane domains, suggesting conserved structural features. These GPCRs include an orphan peptide receptor as well as several with strong sequence homologies with rhabdomeric opsin receptors, a serotonin receptor, a sulfakinin (SK) receptor, an allatostatin-A (buccalin) receptor and an FMRFamide receptor. Buccalin and FMRFa peptides were identified in water conditioned by *B. glabrata*, and we show synthetic buccalin and FMRFa can stimulate significant rates of change of direction and turn-back responses in *S. mansoni* miracidia. Ortholog GPCRs were identified in *S. mansoni* miracidia and *B. glabrata*. These GPCRs may detect similar ligands, including snail-derived odorants that could facilitate miracidial host finding. These results lay the foundation for future research elucidating the mechanisms by which GPCRs mediate host finding which can lead to the potential development of novel anti-schistosome interventions.

## Introduction

Metazoan helminth blood flukes of the genus *Schistosoma* are the primary etiological agents of human schistosomiasis, a disease endemic in 74 countries that affects over 200 million people worldwide^1^. Globally, up to 200,000 people die directly or indirectly due to schistosomiasis annually^2^ and an estimated 600 million people live in endemic areas^3^. Schistosomes have a complex dioecious life cycle, involving asexually reproduced larvae in a molluscan intermediate host and sexually reproductive adult worms in the mammalian definitive host. In water, *Schistosoma mansoni* eggs hatch into free-living, mobile miracidia that must search and infect a compatible snail host, *Biomphalaria glabrata*^4^. Following infection, a single miracidium can reproduce asexually via mother and daughter sporocysts, into several thousand cercariae that each when released may develop into an adult worm in the human host. The complex physiological and morphological changes associated with the *Schistosoma* life-cycle means that the anthropogenic control of transmission could be directed at several life cycle stages. Currently, the adult worms are usually targeted through treatment of infected humans with the drug praziquantel^5^. Nevertheless, the disease remains a constant threat in developing countries and the World Health Organization has acknowledged that continued research on the snail infection stage is required^6^. For example, alternative methods of interfering with the *Schistosome* life-cycle could involve the application of anthelmintic drugs that target the host seeking behavior of miracidia or cercariae^7,8^.

Schistosome miracidia have restricted vision and limited time (12–16 h) to find an appropriate host snail^9^. Host identification involves a range of behavioral responses that promote host localization, thereby increasing the likelihood of successful infection^10,11^. Sharing the same living environment with the intermediate host makes the miracidial stage an ideal point to interrupt the parasite’s life cycle and represents a target window for assessing the molecular components required for host finding. Two main behavioural responses occur comprising an initial dispersal and directional phase influenced by photoreceptor mediation, and a secondary seeking and circling phase (chemokinesis) that is olfactory-mediated^7,12–14^. To date, little information is available concerning the underlying molecular mechanisms that dictate olfactory-mediated host detection by schistosome miracidia although a peptide was recently discovered from *B. glabrata* that induces behavioural changes in miracidia^15^.

G protein-coupled receptors (GPCRs) are well recognized as chemosensory receptors in eumetazoans^16^, and are a promising research focus for understanding parasite host-finding. GPCRs and downstream biochemical pathways are often used as selective pharmacological targets for parasite lifecycle interruption^17,18^, and thus may be effective targets for miracidial manipulation. At the molecular level, there are some reports on *S. mansoni* receptor biology and signal transduction pathways such as the discovery of an array of genome-encoded sensory-type proteins^19,20^. These include GPCRs^21^ that may interact with chemical ligands, a concept supported by proteomic and functional expression analyses that identified GPCRs in the miracidia and adult tegumental matrix of schistosomes^11,22,23^. Scrutiny of the *S. mansoni* genome revealed numerous rhodopsin-type GPCR sequences using a combination of three bioinformatic algorithms, including the Phobius, HMMerSearch and Pfam scan^24,25^. Those expressed in the miracidia included two opsin receptors, which may underpin miracidial photokinetic behavior^26,27^.

Emerging information shows that the close association of the snail and its obligate schistosome parasite has helped in shaping their respective genomes. The genetic variability of the snail host, rather than the human host, may be a more significant factor in influencing the variability of life history traits in schistosomes^28–30^. For example, it has been noted that the presence of homologous mucin proteins between the snail and different strains of *S. mansoni* may be key elements underlying snail host-parasite compatibility^31^. This is consistent with the discovery of significant homology of the non-coding 5′ and 3′ regions of non-long terminal repeat retrotransposon nimbus sequences; these class I transposable elements copy and paste themselves into different genomic location, in the host and parasite, suggesting possible horizontal transfer of host sequences into the parasite^32,33^.

In this study, we identified *S. mansoni* miracidia rhodopsin-like GPCRs that share significant sequence identity with rhodopsin-like GPCRs in *B. glabrata*. This new knowledge guided peptide behaviour bioassays on *S. mansoni* miracidia which demonstrated that FMRFa and buccalin peptides can elicit behaviours consistent with host finding.

## Materials and methods

### Ethics approval and consent to participate

The conduct and procedures involving animal experimentation were approved by the Animal Ethics Committee of the QIMR Berghofer Medical Research Institute (project number P242). This study was performed in accordance with the recommendations in the Guide for the Care and Use of Laboratory Animals of the National Institutes of Health. The study was carried out in compliance with the ARRIVE guidelines.

### S. mansoni miracidia collection and transcriptome preparation

Livers were obtained from ARC Swiss mice infected with *S. mansoni* (Puerto Rican strain) under conditions specified by the Australian Department of Agriculture, Fisheries and Forestry (DAFF). Mice were euthanized with CO2 gas and their livers were perfused with chilled PBS. Eggs of S. mansoni were collected during perfusion of mice. Four infected mouse livers were sliced with scalpel blades and blended to a smooth consistency in 50 mL phosphate buffered saline (PBS). A two-day protocol was used to obtain relatively clean schistosome eggs and miracidia^34^. In brief, the mixture of eggs and mouse liver tissue were incubated with collagenase B, penicillin and streptomycin at 37 °C overnight, followed by fractionation using Percoll columns (8 ml Percoll + 32 ml of 0.25 M sucrose in 50 ml tubes). The egg pellets were washed using PBS twice on a second Percoll column (2.5 ml Percoll + 7.5 ml 0.25 M sucrose in a 15 ml tube). Purified eggs were transferred into a 200 ml hatching measuring cylinder wrapped completely in light-blocking black tape with the exclusion of the top 4 cm from the lip, thereby producing a light-gradient. The hatching cylinder was topped with pH neutral MilliQ water until ~ 1.5 cm above the tape-covered area and exposed to bright light at 27 °C. Eggs were incubated for 6 h post-hatch, and the top 10 ml of miracidium-containing water was collected for miracidia isolation. Hatched miracidia were isolated by centrifugation at 8,000 × g for 1 min at 4 °C, and washed twice with water. For RNA isolation, miracidia were collected at 6 h post-hatch and kept at -80℃. Total RNA was isolated from *S. mansoni* miracidia (6 h post-hatch in triplicate) using TRIzol reagent following the manufacturer’s user guide (Invitrogen, USA), the RNA quantity and quality were assessed by UV spectrophotometry (NanoDrop ND-1000) and the RNA was sent to NovoGene (Hongkong) for next-generation Illumina 2500 platform RNA-seq. Raw sequence data was deposit into the GenBank NCBI under accession number SRR17224866. De novo transcriptome assembly of the *S. mansoni* miracidia raw sequence data was performed using Trinity, as previously described^35,36^ and contigs were translated into protein sequences using Transdecoder^35^. Gene expression levels of the *S. mansoni* miracida were calculated by mapping raw sequence data against the *S. mansoni* reference genome derived from WormBase Parasite (https://parasite.wormbase.org/Schistosoma_mansoni_prjea36577/Info/Index/) using CLC Genomic Workbench with default parameters^37^.

### Gene identification and functional annotation

**Figure 1:**
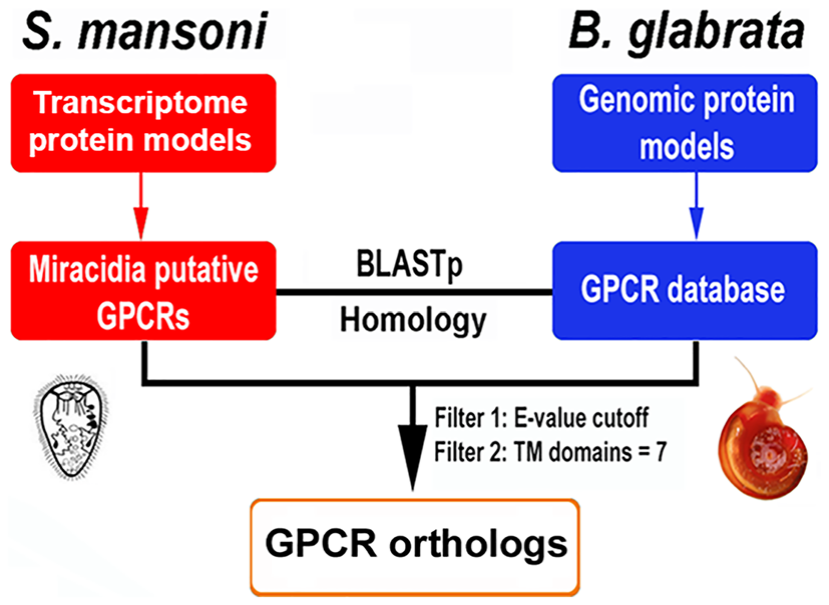
Workflow for identification of shared GPCRs mined from the *B. glabrata* genome and transcriptome of *S. mansoni* miracidia.

The pipeline for identification of ortholog GPCRs shared between *B. glabrata* and *S. mansoni* is shown in Fig. 1. For *B. glabrata,* data on GPCRs and their expression levels in different tissues were retrieved from a previous study^6^. With *S. mansoni*, transcriptome-derived protein sequences were searched for Pfam-based profiles and TM domains to identify receptors that belong to the rhodopsin GPCR family. Specifically, this included two bioinformatic tools to predict TM domains for all proteins, including TMHMM (http://www.cbs.dtu.dk/services/TMHMM-2.0/) and Phobius (http://phobius.sbc.su.se/). As TM domains are convenient markers for GPCRs, we only focused on those sequences with 7-TM domains. Next, we applied a Pfam-based profile search using HMMerSearch (http://www.hmmer.wustl.edu/). Proteins containing putative rhodopsin-type GPCR domains were systematically identified by profile hidden Markov model searches using the HMMer package (http://www.hmmer.wustl.edu/) and the PFAM model PF00001 (7tm_1).

Putative GPCRs identified in *S. mansoni* miracidia were used to query (using tBLASTp) the *B. glabrata* GPCR database. Those sequences with E-values < 1.0E-20 were retrieved and screened for the presence of recurrent transmembrane motifs using TMHMM (http://www.cbs.dtu.dk/services/TMHMM/). Those containing 7 transmembrane (TM) domains were selected for further analysis. Multiple sequence alignments were created with Molecular Evolutionary Genetics Analysis (MEGA) software version 6.0^38^ with the MUSCLE^39^ algorithm. Phylogenetic trees were constructed using the neighbor-joining method with 1000 bootstrap replicates for node support. Gene ontology mapping and functional annotation were applied by using OmicsBox (BioBam)^40^. The final phylogenetic tree and heatmap were modified with iTOL v5^41^.

### Miracidia behaviour in response to test solutions

#### Test solutions

Synthetic FMRFa (FMRF-NH_2_), buccalin (RLDRFGFAGQL-NH_2_) and SK (NYGDYGIGGGRFGR) were provided by China Peptides (Shanghai, China) (purity ≥ 95%). Serotonin (5-hydroxytryptamine/5-HT) was provided by Sigma (Burlington, United States) (purity ≥ 98%). Stock solutions of FMRFa (100 μM), buccalin (100 μM), SK (100 μM) and 5-HT (5 nM) were prepared by dissolving in MilliQ water. MilliQ water was used as a negative control in bioassays.

#### Miracidia collection and assay

Isolation of *S. mansoni* miracidia at ~ 2 h post-hatch was performed as described in Wang et al.^21^. For each assay, 30 ± 5 actively swimming miracidia in pH neutral MilliQ water (~ 4 ml in total) were evenly distributed with a pipette to the central region of a Petri dish (100 mm × 15 mm) containing 4 ml of MilliQ water. The swimming area for the miracidia was covered to prevent light bias prior to analysis under the microscope. For assays, 2 µl of test solution were added to the central area of the Petri dish. Some diffusion of each molecule was expected over the 1 min test period. Assays were also tested at 10 × and 100 × serial dilutions. To record miracidia movement before and after addition of the test solutions, an inverted compound microscope with videoing capacity (OLYMPUS CKX41), fitted with an OLMPUS DPI Digital Microscope Camera DP22 (2.8-megapixel image at a rate of 15 frames per second), was used. The real camera’s field of view (FOV) was 2.500 × 1.875 mm. Miracidia movement was recorded for 1 min before and after the addition of each test solution and captured videos were processed using Tracker 4.87.

#### Analysis

Miracidia trajectories were tracked manually from entrance into the FOV to exit, or up to 1 min for those that remained within the FOV. Only miracidia that had been swimming for more than the length of the short edge (7.5 cm) of the FOV were included before and after solution addition (File S1); those that did not were considered statistically meaningless. The average time duration of miracidia staying within the FOV was considered as another key behavioural feature and was statistically compared. For those miracidia staying for more than 1 min after addition of solution, the time duration within that 1 min was used for comparison, and the mean acceleration value was calculated. Miracidium acceleration and velocity were calculated based on trajectories, with units converted to cm s^-2^ and cm/s. A paired two-tailed t-test was used to calculate *P*-values; in addition, wherever applicable, two-way ANOVA analysis^42^ was performed to evaluate the significance of changes (acceleration, velocity and time) among the test solutions and the negative control.

### Identification of candidate peptide ligands from B. glabrata-conditioned water extract

To identify whether candidate peptide ligands were present in *B. glabrata*-conditioned water, two approaches were taken. First, mass spectrometry data derived from prior analysis of *B. glabrata*-conditioned water^43^ was searched using target precursor proteins.

Second, antibody-mediated dot blot analysis was performed using *B. glabrata*-conditioned water extracts. *B. glabrata* were washed with pH neutral MilliQ water and placed in beakers containing 20 ml water for 2 h at room temperature (RT). Snails were removed, conditioned water was collected from 20 snails (in different aquaria), and 20 ml methanol was added to each beaker and mixed thoroughly. The conditioned water was filtered through PVDF Millex-HV syringe filter units (0.45 µm) to remove particles and microbes. The filtrate was snap frozen and lyophilized. For negative controls, water not previously exposed to snails was similarly processed. When required for dot bot assay, samples were rehydrated with 200 μl MilliQ water and centrifuged at 12,000 rpm for 5 min. The supernatant was collected and diluted 1:1 in MilliQ water. Quantitation was performed using a NanoDrop 2000c UV–Vis spectrophotometer (Thermo Scientific, Waltham, U.S) at A280nm. *B. glabrata* extracts at 5 g$\upmu$/$\upmu$l, 500 ng/$\upmu$l and 50 ng/$\upmu$l were applied (2 μl) onto a nitrocellulose membrane (0.45 m$\upmu$, BioRad, Hercules, U.S) that had been pre-soaked in 1 × phosphate buffered saline (PBS) and air-dried at room temperature for 10 min. The membrane was incubated in blocking buffer (2% (v/v) skim milk in PBS) for 1 h, and primary antibody was added [1:500; rabbit anti-buccalin^44^, rabbit anti-FMRFa (Genscript, Piscataway, U.S), rat anti-5-HT (Sigma)] for 1 h at RT. Membranes were washed with PBS-Tween20 (0.1% (v/v)) and incubated for 1 h at RT with secondary antibody [1:5000; anti-rabbit Ig-IR 680 (LI-COR, Lincoln, U.S) or anti-rat Ig-IR 795 (LI-COR)]. Following washes (3x, 5 min) in PBST, blots were visualized using an Odyssey CLx, LI-COR machine.

### Availability of data and materials

The *S. mansoni* miracidial transcriptomic raw sequence data was deposit into the GenBank NCBI under accession number SRR17224866 (https://www.ncbi.nlm.nih.gov/sra/?term=SRR17224866). The *S. mansoni* reference genome is availabe from WormBase Parasite (https://parasite.wormbase.org/Schistosoma_mansoni_prjea36577/Info/Index/). The original *B. glabrata* protein dataset is available from the VectorBase(https://vectorbase.org/vectorbase/app/downloads/Current_Release/BglabrataBB02/fasta/data/).

## Results

### Identification of ortholog GPCRs shared between B. glabrata and S. mansoni miracidia

**Figure 2:**
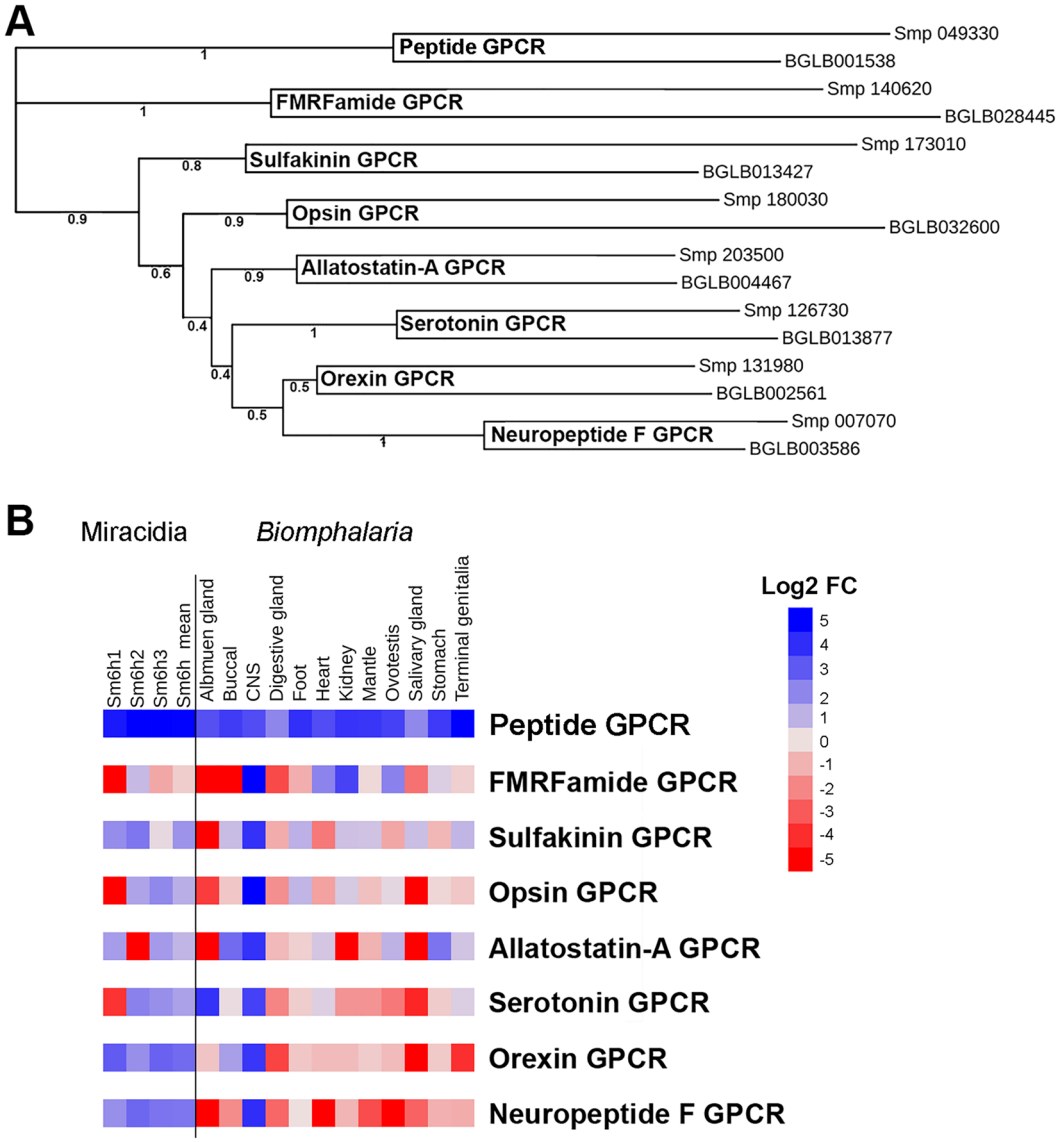
Characterization of GPCR orthologs shared between *B. glabrata* and *S. mansoni* miracidia. (**A**) Phylogenetic tree analysis of shared GPCRs, where each *B. glabrata* GPCR clusters with an ortholog receptor from *S. mansoni*. Bootstrap values support the confidence levels of clades. (**B**) Heatmap showing expression of shared GPCR- (in TPM) encoding genes in *S. mansoni* miracidia at 6 h post-hatch and different *B. glabrata* tissues. The columns represent *S. mansoni* miracidia biological replicates (1–3) and their mean, as well as *Biomphalaria* (*glabrata*) tissues.

In total, 96 proteins with 7-TM domains were extracted from the *S. mansoni* transcriptome-derived protein models based on TMHMM prediction. Phobius prediction led to the identification of 139 proteins with 7-TM domains. Pfam profiling of both predictions led to the classification of 87 proteins (E-value < 0.0004) as rhodopsin-type receptors. BLASTp analysis of these GPCRs against all *B. glabrata* rhodopsin GPCRs showed significant matches (E-value < 1.0E−20) for 8 GPCRs (Table 1), with between 26 to 48% amino acid identity (File S2a). BLASTp searches using all *S. mansoni* and *B. glabrata* ortholog GPCRs against the NCBI non-redundant (nr) database returned best match hits for GPCRs, including 5-HT (serotonin), allatostatin-type A (AST-A), FMRFa, SK, orexin type 2, neuropeptide F, orphan peptide and opsin-like GPCRs (Table 1, Fig. 2A and File S2b). In *B. glabrata*, all GPCRs were expressed in the CNS, while the orphan rhodopsin GPCR had the widest tissue distribution (Fig. 2B). In *S. mansoni* miracidia, the rhodpopsin GPCR ortholog was found to show the highest average level of expression, compared to other receptors (Fig. 2B).

**Table 1:** Comparative sequence identity between *Biomphalaria glabrata* and *Schistosoma mansoni* GPCR homologs.

*S. mansoni* ID	*B. glabrata* ID	E-value	Overall identity (%)	TM number (% identity)	Best match GPCR
Smp_140620	BGLB028445	2.78E-50	26.8	1 (45.5), 2 (64.0), 3 (58.3), 4 (40.0), 5 (39.1), 6 (45.8), 7 (63.2)	FMRFa
Smp_131980	BGLB002561	2.88E-42	30.5	1 (40.0), 2 (40.0), 3 (76.2), 4 (50.0), 5 (68.4), 6 (70.8), 7 (69.6)	Orexin type 2
Smp_173010	BGLB013427	2.67E-23	48.5	1 (45.6), 2 (60.0), 3 (45.8), 4 (52.0), 5 (39.1), 6 (50.0), 7 (63.2)	Sulfakinin
Smp_007070	BGLB003586	2.69E-60	31.2	1 (29.2), 2 (41.7), 3 (42.9), 4 (27.8), 5 (36.8), 6 (56.5), 7 (60.9)	Neuropeptide F
Smp_126730	BGLB013877	9.83E-37	34.5	1 (27.3), 2 (26.1), 3 (61.9), 4 (66.7), 5 (34.8), 6 (60.9), 7 (60.9)	Serotonin
Smp_180030	BGLB032600	4.18E-20	24.9	1 (52.2), 2 (68.2),3 (74.9), 4 (52.6),5 (38.1), 6 (66.7), 7 (85.0)	Opsin
Smp_203500	BGLB004467	2.08E-44	30.1	1 (47.8), 2 (34.8), 3 (37.0), 4 (34.8), 5 (47.6), 6 (62.5), 7 (58.3)	Allatostatin A/buccalin
Smp_049330	BGLB001538	1.44E-59	36.8	1 (42.8), 2 (54.1), 3 (32.8), 4 (39.4), 5 (41.6), 6 (64.5), 7 (54.7)	Rhodopsin

### Bioassay of putative ligands on S. mansoni miracidia behaviour

**Figure 3:**
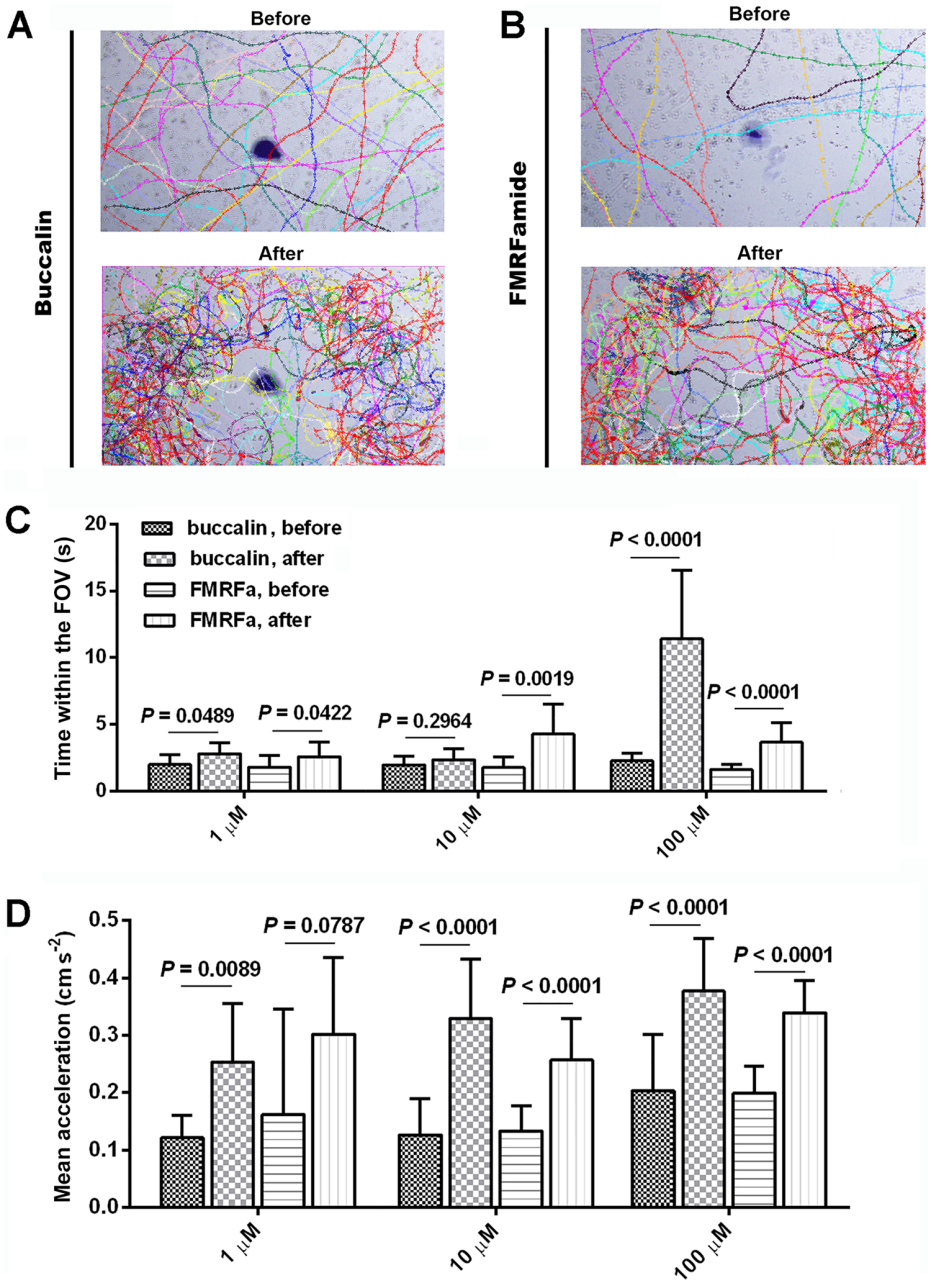
Miracidial behaviour assay using buccalin and FMRFa peptides. (**A**) Representative trajectories of miracidial movement before and after the addition of the buccalin solution (100 µM) to the center of the recording area. Each colour represents one indistinguishable miracidium individual. See Movie S1 for representative assay video. (**B**) Representative trajectories of miracidia movement before and after the addition of the FMRFa solution (100 µM) to the center of the area. Each colour represents one indistinguishable miracidium individual. See Movie S2 for representative assay video. (**C**) Graph of time duration of miracidia remaining in the videoing zone, and (**D**) mean acceleration values, before and after the addition of buccalin and FMRFa.

The elucidation of interspecies GPCR orthologs with putative ligands presented an opportunity to investigate how snail-derived biomolecules influence *S. mansoni* miracidia. The 5-HT is a well-established eumetazoan neurotransmitter, while and *B. glabrata* neuropeptides FMRFa, buccalin [recognised as AST-A homolog in molluscs^45^] and SK have previously been identified in *B. glabrata*^6,46^, so were tested for bioactivity on miracidia. The trajectories of miracidial movement before and after addition of the FMRFa and buccalin peptides (2 µl at 100 µM) are compared in Fig. 3A,B. Representative movies can be viewed in Movies S1 and S2. Before addition, miracidia generally swam across the FOV in a direct, linear path (Fig. 3A,B—Before). Following application, miracidia showed localized movement within the FOV, as well as increased circular swimming (Fig. 3A,B—After). Upon application of buccalin or FMRFa peptides, miracidia within the FOV swam for longer, except for buccalin at 10 µM (*P*-value = 0.2964) (Fig. 3C). The change in acceleration were significant after the addition of buccalin or FMRFa (100 µM and 10 µM) (Fig. 3D). Peptides stimulated a swimming pattern concentrated around the location of the peptides.

In contrast, following application of 5-HT (5 nM; Movie S3), the time within the FOV was insignificant, yet the change of acceleration was significant (File S3). Peptides (100 µM) resulted in miracidia staying longer in the region where the peptide was added, but there was no significant change in average velocity, as indicated by two-way ANOVA analysis (File S4). There was also no significant change in the time within FOV, nor in average velocity, upon addition of the SK peptide at 3 different concentrations (100 µM, 10 µM and 1 µM; Movie S4). As negative controls, MilliQ water was tested on *S. mansomi* miracidia and no behavior changes was observed.

### Detection of buccalin and FMRFa peptides in B. glabrata-conditioned water

**Figure 4:**
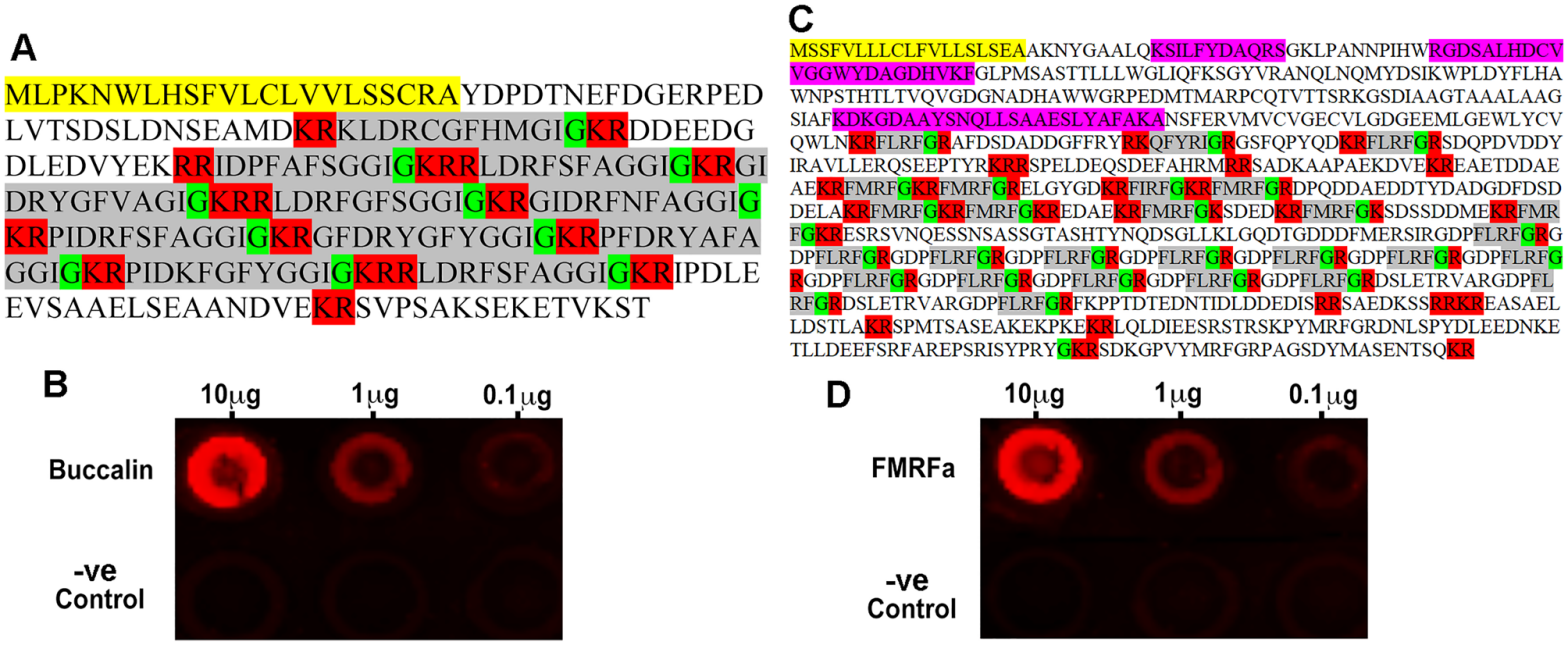
*Biomphalaria glabrata* precursor sequence and dot blot assay for buccalin and FMRFa in *B. glabrata*-conditioned water. (**A**) Buccalin neuropeptide precursor sequence (**B**) Dot blot using anti-buccalin on *B. glabrata*-conditioned water extracts at 10, 1 and 0.1 mg. (**C**) Buccalin neuropeptide precursor sequence. (**D**) Dot blot using anti-FMRFa on *B. glabrata*-conditioned water extracts at 10, 1 and 0.1 g$\upmu$. -ve Control represents purified water extract only. For precursor sequences, yellow—signal peptide, red—putative cleavage sites, green—amidation, grey—bioactive peptides, pink—MS peptide matches.

The *B. glabrata* buccalin precursor contains numerous buccalin-like peptides (Fig. 4A). The most conserved region in the buccalins is a C-terminal FXGGIG, which following post-translational processing, becomes an amidated peptide. Dot blots performed on conditioned-water extracts from *B. glabrata* showed the presence of a buccalin-like peptide at extract dilutions from 10 μg to 0.1 μg (Fig. 4B). The *B. glabrata* FMRFa precursor contains numerous FMRFa related peptides (FaRPs), including FMRFa, FLRFa and FIRFa (Fig. 4C). Analysis of proteomic mass spectrometry data derived from naïve *B. glabrata* snail-conditioned water^43^, identified 4 peptide matches for the FMRFa precursor, although not specifically within any FaRPs. Dot blots performed on conditioned-water extracts from *B. glabrata* showed the presence of peptide(s) with similarity to FaRPs at extract dilutions from 10 μg to 0.1 μg (Fig. 4D).

## Discussion

*S. mansoni* miracidia respond to snail-derived biomolecules^43^, although the precise identity of the active biomolecule(s) has not been clearly defined. One study implicated “miracidia-attracting glycoproteins” present within the snail mucus^12^, while in silico analysis from *B. glabrata* snail conditioned-water proteins predicted interactions of uncharacterized *S. mansoni* proteins with *B. glabrata* proteins^43^. Peptides have also been implicated, whereby a snail-derived novel peptide (named P12) stimulated changes in the behaviour of the *S. mansoni miracidia*^47^.

In this study, to narrow down biomolecules potentially involved in the parasite and host interplay, we utilised gene resources from both the *B. glabrata* and *S. mansoni* to identify ortholog GPCRs that are likely used by each organism to detect similar ligands. We reported that 8 *S. mansoni* miracidia GPCRs share significant identity with 8 *B. glabrata* GPCRs, not only in GO mapping, but also within regions corresponding to putative TM domains. These include GPCRs with similarity to neuropeptide GPCRs that bind FMRFa, AST-A/buccalin and sulfakinin peptides. We propose that the miracidial ortholog GPCRs may be used for neural signaling, requiring a common ligand, and/or are used to detect semiochemical biomolecules present within the water. The latter expectation was validated by miracidial behaviour changes in the presence of snail FMRFamide and AST-A/buccalin peptides.

The AST-A and its receptor have been characterised in various insects^48^ where they are involved in multiple functions such as inhibition of juvenile hormone biosynthesis and reduction of food intake^48^, AST-A-like neuropeptides have been identified in gastropods and bivalve molluscs, including *Lottia gigantea*, *Theba pisana, Aplysia californica* and *Crassostrea gigas*^49–52^. Buccalin, named following its first identification in the accessory radula closer muscle of *A. californica*^53^, has been implicated in various activities in molluscs such as the inhibition of muscle contraction, regulation of feeding and spawning^53–55^. Also in gastropods and bivalves, the AST-A/buccalin receptor was identified through in silico analysis of publicly available genomic datasets including that of *B. glabrata*^56^. In our study, we identified an AST-A/buccalin receptor ortholog in *S. mansoni*, although there are no reports that *S. mansoni* has a buccalin-like peptide. In fact, a comprehensive neuropeptide investigation of 10 platyhelminth species showed that only the free-living turbellarian *Macrostomum lignano* has a buccalin-like peptide (*npp-9* gene; GAYSGFLG)^57^. We identified a buccalin-like peptide in the *B. glabrata* conditioned water. Despite the presence of neuropeptides in mucus secretions having not been well investigated, we previously identified neuropeptides (including buccalin) within the salivary gland mucus of the land snail *T. pisana*^58,59^.

The FMRFa was first discovered in the hard clam *Mercenaria mercenaria* and it is thought to have a pleitropic role in molluscan physiology^60–63^. Extensive studies performed on the freshwater snail *Helisoma* showed that FMRFa and related peptides are densely concentrated not only in the nervous system but also within the salivary glands^64^. An FMRFa receptor has been identified in the heart and nervous tissue of the land snail *Helix*^62,65^ and the optic lobe membrane of the squid *Loligo pelei*^66^. The *S. mansoni* genome contains a gene encoding a FLP precursor (*npp-13* gene) that may be processed to release two RFamide peptides (HFMPQRFa and YTRFVPQRFa)^57^. A synthetic FLP (GNFFRFa) derived from non-schistosome platyhelminth precursors stimulates contraction of *S. mansoni* muscle fibres in vitro^67^. An FLP receptor has also been reported in the turbellarian flatworm *Girardia tigrina* based on sequence similarity and a receptor calcium mobilization assay^68^. The homolog receptor in *S. mansoni* miracidia was investigated in the current study due to its similarity with the *B. glabrata* FMRFa receptor. Our behaviour assays also indicated that snail-derived FMRFa can be detected by *S. mansoni* miracidia due to their staying significantly longer in FOV and the increased acceleration of miracidia, supported by the observed presence of FMRFa precursor peptides in *B. glabrata* conditioned water. However, as *S. mansoni* has the potential to generate endogenous FLPs, we cannot preclude the possibility that the applied FMRFa may stimulate endogenous effects, leading to the observed miracidial behaviour changes.

The monoamine 5-HT plays a critical role in neural transmission and has been very well documented throughout eumetazoans, as has the conservation of 5-HT GPCRs. In adult *S. mansoni,* 5-HT stimulates motor activity^69^, while in the miracidia, an immunofluorescent approach localized 5-HT to within sensory nerves^70^. The 5-HT GPCR was identified within our interspecies GPCR ortholog analysis yet we found that 5-HT at 5 mM did not modify miracidial behaviour, while the significant change in acceleration could be attributed to its high concentration.

Sulfakinin is a sulfated neuropeptide best known for its function as a satiety (food intake) factor^71^. In silico data-mining showed that molluscan SK has the C-terminal RF(W)amide sequence common to insect sulfakinins, as well as the DY motif shared by both insect SKs and vertebrate cholecystokinin (CCK)^72^. Since vertebrate CCKs and insect SKs reveal similar biological function relating to digestive enzyme secretion, satiety and smooth muscle contraction^73^, it is possible that their molluscan counterparts have retained similar basic biological activities. In contrast, there is no obvious SK in *S. mansoni,* suggesting that the parasite may only recognize the *B. glabrata* SK, either as a secreted semiochemical, or once it penetrates the snail as a guidance peptide to navigate to the hepatopancreas where it proliferates^74^. Our behavior assays demonstrated that SK did not alter miracidial behavior (neither the velocity nor duration present under FOV were affected), and therefore it is more likely to act as an internal stimulus in *S. mansoni*.

FMRFa and buccalin peptides may contribute to a cocktail of biomolecules that could be used as an effective, species-specific attractant. Our serial dilution assays suggested sustained bioactivity for both buccalin and FMRFa peptides at a concentration of at least 1 µM. We also report 1 orphan peptide GPCR ortholog within *B. glabrata* and *S. mansoni* miracida, which is consistent with the possibility that uncharacterized species-specific peptides could help attract miracidia to the appropriate snail host due to its presence in many tissues of *B. glabrata* and its high expression level in *S. mansoni* miracidia.

## Conclusions

To minimise transmission and reduce schistosomiasis prevalence, interference with the snail-miracidium interaction is a promising plan of biocontrol. We characterised ortholog GPCRs shared between *B. glabrata* and *S. mansoni* miracidia, important biomolecules commonly used for chemosensory communication. The *B. glabrata* buccalin and FMRFa GPCRs represented good targets for bioassay, the results from which indicated that buccalin and FMRFa stimulated miracidial behaviour changes, despite the fact that homologs of buccalin-like peptides are not present in *S. mansoni*. These GPCRs could present novel targets for the development of anti-helminthic compounds to be applied to lakes and specifically interfere with Schistosoma detection of snail host. For greater species-specificity, we suggest that deorphanizing the ortholog orphan peptide GPCR will be most advantageous. These findings further help our understanding of chemosensory interaction between parasites and their hosts, particularly within aquatic environments.

## Supplementary Information

Supplementary Information 1.

Supplementary Information 2.

Supplementary Information 3.

Supplementary Information 4.

Supplementary Information 5.

Supplementary Information 6.

Supplementary Video 1.

Supplementary Video 2.

Supplementary Video 3.

Supplementary Video 4.

## Abbreviations

GPCRs: G protein-coupled receptors
HMMs: Hidden Markov Models
MEGA: Molecular evolutionary genetics analysis
PBS: Phosphate buffered saline
PBST: PBS with 0.1% Tween 20
DAPI: 4,6-Diamidino-2-phenylindole
PFA: Paraformaldehyde
FOV: Field of view
serotonin: 5-Hydroxytryptamine
SK: Sulfakinin
CNS: Central nervous system
MS: Mass spectrometry
USC: University of the sunshine coast
QIMR: Queensland Institute of Medical Research Institute

## References

[1] McManus DP, Dunne DW, Sacko M, Utzinger J, Vennervald BJ, Zhou XN (2018). Schistosomiasis. Nat. Rev. Disease Prim..

[2] Lozano R, Naghavi M, Foreman K, Lim S, Shibuya K, Aboyans V, Abraham J, Adair T, Aggarwal R, Ahn SY (2013). Global and regional mortality from 235 causes of death for 20 age groups in 1990 and 2010: A systematic analysis for the Global Burden of Disease Study 2010. The Lancet.

[3] Thétiot-Laurent SAL, Boissier J, Robert A, Meunier B (2013). Schistosomiasis chemotherapy. Angew. Chem. Int. Ed..

[4] Ittiprasert, W., Myers, J., Odoemelam, E. C., Raghavan, N., Lewis, F., Bridger, J. M., & Knight, M. Advances in the Genomics and Proteomics of the Freshwater Intermediate Snail Host of Schistosoma mansoni, Biomphalaria glabrata. In: *Biomphalaria Snails and Larval Trematodes.* edn.: Springer; 2011: 191–213.

[5] Berriman M, Haas BJ, LoVerde PT, Wilson RA, Dillon GP, Cerqueira GC, Mashiyama ST, Al-Lazikani B, Andrade LF, Ashton PD (2009). The genome of the blood fluke Schistosoma mansoni. Nature.

[6] Adema CM, Hillier LW, Jones CS, Loker ES, Knight M, Minx P, Oliveira G, Raghavan N, Shedlock A (2017). Whole genome analysis of a schistosomiasis-transmitting freshwater snail. Nat. Commun..

[7] Haberl B, Kalbe M, Fuchs H, Ströbel M, Schmalfuss G, Haas W (1995). Schistosoma mansoni and S. haematobium: Miracidial host-finding behaviour is stimulated by macromolecules. Int. J. Parasitol..

[8] Haas W, Gui M, Haberl B, Strobel M (1991). Miracidia of Schistosoma japonicum: approach and attachment to the snail host. J. Parasitol..

[9] Sobhon, P., & Upatham, E. S. Snail hosts, life-cycle, and tegumental structure of oriental schistosomes. *Snail hosts, life-cycle, and tegumental structure of oriental schistosomes* 1990.

[10] Boissier J, Mone H (2001). Male-female larval interactions in Schistosoma mansoni-infected Biomphalaria glabrata. Int. J. Parasitol..

[11] Campos TD, Young ND, Korhonen PK, Hall RS, Mangiola S, Lonie A, Gasser RB (2014). Identification of G protein-coupled receptors in Schistosoma haematobium and S. mansoni by comparative genomics. Parasit. Vect..

[12] Kalbe M, Haberl B, Haas W (2000). Snail host finding by Fasciola hepatica and Trichobilharzia ocellata: Compound analysis of “miracidia-attracting glycoproteins”. Exp. Parasitol..

[13] Chaisson KE, Hallem EA (2012). Chemosensory behaviors of parasites. Trends Parasitol..

[14] Hertel J, Holweg A, Haberl B, Kalbe M, Haas W (2006). Snail odour-clouds: spreading and contribution to the transmission success of Trichobilharzia ocellata (Trematoda, Digenea) miracidia. Oecologia.

[15] Sakmar TP (1998). Rhodopsin: A prototypical G protein-coupled receptor. Prog. Nucl. Acid Res. Mol. Biol..

[16] Doty RL (2015). Handbook of Olfaction and Gustation.

[17] Fitzpatrick JM, Peak E, Perally S, Chalmers IW, Barrett J, Yoshino TP, Ivens AC, Hoffmann KF (2009). Anti-schistosomal intervention targets identified by lifecycle transcriptomic analyses. PLoS Negl. Trop. Dis..

[18] Taft AS, Norante FA, Yoshino TP (2010). The identification of inhibitors of Schistosoma mansoni miracidial transformation by incorporating a medium-throughput small-molecule screen. Exp. Parasitol..

[19] Hoffmann KF, Davis EM, Fischer ER, Wynn TA (2001). The guanine protein coupled receptor rhodopsin is developmentally regulated in the free-living stages of Schistosoma mansoni. Mol. Biochem. Parasitol..

[20] Dissous C, Morel M, Vanderstraete M (2014). Venus kinase receptors: prospects in signaling and biological functions of these invertebrate kinases. Front. Endocrinol. (Lausanne).

[21] Consortium SjGSaFA (2009). The *Schistosoma japonicum* genome reveals features of host-parasite interplay. Nature.

[22] El-Shehabi F, Vermeire JJ, Yoshino TP, Ribeiro P (2009). Developmental expression analysis and immunolocalization of a biogenic amine receptor in Schistosoma mansoni. Exp. Parasitol..

[23] El-Shehabi F, Taman A, Moali LS, El-Sakkary N, Ribeiro P (2012). A novel G protein-coupled receptor of Schistosoma mansoni (SmGPR-3) is activated by dopamine and is widely expressed in the nervous system. PLoS Negl. Trop. Dis..

[24] Liang D, Zhao M, Wang T, McManus DP, Cummins SF (2016). GPCR and IR genes in Schistosoma mansoni miracidia. Parasit Vect..

[25] Zamanian M, Kimber MJ, McVeigh P, Carlson SA, Maule AG, Day TA (2011). The repertoire of G protein-coupled receptors in the human parasite Schistosoma mansoni and the model organism Schmidtea mediterranea. BMC Genom..

[26] Mason PR, Fripp PJ (1977). The reactions of Schistosoma mansoni miracidia to light. J. Parasitol..

[27] Short RB, Gagne HT (1975). Fine structure of possible photoreceptor in cercariae of Schistosoma mansoni. J. Parasitol..

[28] Lewis FA, Patterson CN, Knight M, Richards CS (2001). The relationship between Schistosoma mansoni and Biomphalaria glabrata: Genetic and molecular approaches. Parasitology.

[29] Sandland GJ, Foster AV, Zavodna M, Minchella DJ (2007). Interplay between host genetic variation and parasite transmission in the Biomphalaria glabrata-Schistosoma mansoni system. Parasitol Res..

[30] Zavodna M, Sandland GJ, Minchella DJ (2008). Effects of intermediate host genetic background on parasite transmission dynamics: a case study using Schistosoma mansoni. Exp. Parasitol..

[31] Roger E, Gourbal B, Grunau C, Pierce RJ, Galinier R, Mitta G (2008). Expression analysis of highly polymorphic mucin proteins (Sm PoMuc) from the parasite Schistosoma mansoni. Mol. Biochem. Parasitol..

[32] Raghavan N, Tettelin H, Miller A, Hostetler J, Tallon L, Knight M (2007). Nimbus (BgI): an active non-LTR retrotransposon of the Schistosoma mansoni snail host Biomphalaria glabrata. Int. J. Parasitol..

[33] Toledo R, Fried B (2010). Biomphalaria Snails and Larval Trematodes.

[34] Wang T, Zhao M, Rotgans BA, Strong A, Liang D, Ni G, Limpanont Y, Ramasoota P, McManus DP, Cummins SF (2016). Proteomic analysis of the schistosoma mansoni miracidium. PLoS ONE.

[35] Haas BJ, Papanicolaou A, Yassour M, Grabherr M, Blood PD, Bowden J, Couger MB, Eccles D, Li B, Lieber M (2013). De novo transcript sequence reconstruction from RNA-seq using the Trinity platform for reference generation and analysis. Nat. Protoc..

[36] Grabherr MG, Haas BJ, Yassour M, Levin JZ, Thompson DA, Amit I, Adiconis X, Fan L, Raychowdhury R, Zeng Q (2011). Full-length transcriptome assembly from RNA-Seq data without a reference genome. Nat. Biotechnol..

[37] Mortazavi A, Williams BA, McCue K, Schaeffer L, Wold B (2008). Mapping and quantifying mammalian transcriptomes by RNA-Seq. Nat. Methods.

[38] Kumar S, Stecher G, Peterson D, Tamura K (2012). MEGA-CC: computing core of molecular evolutionary genetics analysis program for automated and iterative data analysis. Bioinformatics.

[39] Edgar RC (2004). MUSCLE: Multiple sequence alignment with high accuracy and high throughput. Nucl. Acids Res..

[40] Götz S, García-Gómez JM, Terol J, Williams TD, Nagaraj SH, Nueda MJ, Robles M, Talón M, Dopazo J, Conesa A (2008). High-throughput functional annotation and data mining with the Blast2GO suite. Nucl. Acids Res..

[41] Letunic I, Bork P (2021). Interactive Tree Of Life (iTOL) v5: an online tool for phylogenetic tree display and annotation. Nucl. Acids Res..

[42] Fujikoshi Y (1993). Two-way ANOVA models with unbalanced data. Discret. Math..

[43] Fogarty CE, Zhao M, McManus DP, Duke MG, Cummins SF, Wang T (2019). Comparative study of excretory-secretory proteins released by Schistosoma mansoni-resistant, susceptible and naïve Biomphalaria glabrata. Parasit Vect..

[44] Adamson KJ, Wang T, Rotgans B, Kruangkum T, Kuballa AV, Storey KB, Cummins SF (2015). Genes and associated peptides involved with aestivation in a land snail. Gen. Comp. Endocrinol..

[45] Li Z, Cardoso JCR, Peng M, Inácio JPS, Power DM (2021). Evolution and potential function in molluscs of neuropeptide and receptor homologues of the insect allatostatins. Front. Endocrinol..

[46] Wang T, Zhao M, Liang D, Bose U, Kaur S, McManus DP, Cummins SF (2017). Changes in the neuropeptide content of Biomphalaria ganglia nervous system following Schistosoma infection. Parasit Vect..

[47] Wang T, Wyeth RC, Liang D, Bose U, Ni G, McManus DP, Cummins SF (2019). A Biomphalaria glabrata peptide that stimulates significant behaviour modifications in aquatic free-living Schistosoma mansoni miracidia. PLoS Negl. Trop. Dis..

[48] Stay B, Tobe SS (2007). The role of allatostatins in juvenile hormone synthesis in insects and crustaceans. Annu. Rev. Entomol..

[49] Adamson KJ, Wang T, Zhao M, Bell F, Kuballa AV, Storey KB, Cummins SF (2015). Molecular insights into land snail neuropeptides through transcriptome and comparative gene analysis. BMC Genom..

[50] Veenstra JA (2010). Neurohormones and neuropeptides encoded by the genome of Lottia gigantea, with reference to other mollusks and insects. Gen. Comput. Endocrinol..

[51] Bauknecht P, Jekely G (2015). Large-scale combinatorial deorphanization of platynereis neuropeptide GPCRs. Cell Rep..

[52] Stewart MJ, Favrel P, Rotgans BA, Wang T, Zhao M, Sohail M, O’Connor WA, Elizur A, Henry J, Cummins SF (2014). Neuropeptides encoded by the genomes of the Akoya pearl oyster Pinctata fucata and Pacific oyster Crassostrea gigas: a bioinformatic and peptidomic survey. BMC Genom..

[53] Cropper EC, Miller MW, Tenenbaum R, Kolks MA, Kupfermann I, Weiss KR (1988). Structure and action of buccalin: A modulatory neuropeptide localized to an identified small cardioactive peptide-containing cholinergic motor neuron of Aplysia californica. Proc. Natl. Acad. Sci. USA.

[54] Van In V, Ntalamagka N, O’Connor W, Wang T, Powell D, Cummins SF, Elizur A (2016). Reproductive neuropeptides that stimulate spawning in the Sydney Rock Oyster (Saccostrea glomerata). Peptides.

[55] Miller MW, Beushausen S, Cropper EC, Eisinger K, Stamm S, Vilim FS, Vitek A, Zajc A, Kupfermann I, Brosius J (1993). The buccalin-related neuropeptides: isolation and characterization of an Aplysia cDNA clone encoding a family of peptide cotransmitters. J. Neurosci..

[56] Cardoso JC, Félix RC, Bjärnmark N, Power DM (2016). Allatostatin-type A, kisspeptin and galanin GPCRs and putative ligands as candidate regulatory factors of mantle function. Mar. Genomics.

[57] McVeigh P, Mair GR, Atkinson L, Ladurner P, Zamanian M, Novozhilova E, Marks NJ, Day TA, Maule AG (2009). Discovery of multiple neuropeptide families in the phylum Platyhelminthes. Int. J. Parasitol..

[58] Adamson KJ, Wang T, Rotgans BA, Kuballa AV, Storey KB, Cummins SF (2016). Differential peptide expression in the central nervous system of the land snail Theba pisana, between active and aestivated. Peptides.

[59] Stewart MJ, Wang T, Koene JM, Storey KB, Cummins SF (2016). A "Love" Dart Allohormone Identified in the Mucous Glands of Hermaphroditic Land Snails. J. Biol. Chem..

[60] Lopez-Vera E, Aguilar MB (2008). Heimer de la Cotera EP: FMRFamide and related peptides in the phylum mollusca. Peptides.

[61] Price DA, Greenberg MJ (1977). Structure of a molluscan cardioexcitatory neuropeptide. Science.

[62] Payza K (1987). FMRFamide receptors in Helix aspersa. Peptides.

[63] Voigt KH, Kiehling C, Frosch D, Schiebe M, Martin R (1981). Enkephalin-related peptides: direct action on the octopus heart. Neurosci. Lett..

[64] Bulloch AG, Price DA, Murphy AD, Lee TD, Bowes HN (1988). FMRFamide peptides in Helisoma: identification and physiological actions at a peripheral synapse. J. Neurosci..

[65] Payza K, Greenberg MJ, Price DA (1989). Further characterization of Helix FMRFamide receptors: kinetics, tissue distribution, and interactions with the endogenous heptapeptides. Peptides.

[66] Chin GJ, Payza K, Price DA, Greenberg MJ, Doble KE (1994). Characterization and solubilization of the FMRFamide receptor of squid. Biol. Bull..

[67] Day TA, Maule AG, Shaw C, Halton DW, Moore S, Bennett JL, Pax RA (1994). Platyhelminth FMRFamide-related peptides (FaRPs) contract Schistosoma mansoni (Trematoda: Digenea) muscle fibres in vitro. Parasitology.

[68] Omar HH, Humphries JE, Larsen MJ, Kubiak TM, Geary TG, Maule AG, Kimber MJ, Day TA (2007). Identification of a platyhelminth neuropeptide receptor. Int. J. Parasitol..

[69] Barker LR, Bueding E, Timms AR (1966). The possible role of acetylcholine in Schistosoma mansoni. Br. J. Pharmacol. Chemother..

[70] Jones, B. R., Pan, S. C., & Amarin, M. M. Structure of “Sensory” Nerves and Serotonin-Like Immunofluorescent Activity in Schistosoma Mansoni Miracidia and Cercariae. In: *Biodeterioration Research 2.* edn.: Springer; 1989: 601–626.

[71] Wicher D, Derst C, Gautier H, Lapied B, Heinemann S, Agricola H-J (2007). The satiety signaling neuropeptide perisulfakinin inhibits the activity of central neurons promoting general activity. Front. Cell. Neurosci..

[72] Zatylny-Gaudin C, Favrel P (2014). Diversity of the RFamide Peptide Family in Mollusks. Front. Endocrinol..

[73] Nachman RJ, Giard W, Favrel P, Suresh T, Sreekumar S, Holman GM (1997). Insect Myosuppressins and Sulfakinins Stimulate Release of the Digestive Enzyme α-Amylase in Two Invertebrates: The Scallop Pecten maximus and Insect Rhynchophorus ferrugineus. Ann. N. Y. Acad. Sci..

[74] Gérard C, Moné H, Théron A (1993). Schistosoma mansoni – Biomphalaria glabrata: dynamics of the sporocyst population in relation to the miracidial dose and the host size. Can. J. Zool..

