# Supplementary material for: Analysis of rhodopsin G protein-coupled receptor orthologs reveals semiochemical peptides for parasite (Schistosoma mansoni) and host (Biomphalaria glabrata) interplay
Source: Sci Rep. 2022 May 17;12:8243. doi: 10.1038/s41598-022-11996-x (PMC9114394; doi:10.1038/s41598-022-11996-x)
Supplement: Supplementary file 1 — Supplementary Information 1. [file 41598_2022_11996_MOESM1_ESM.docx]

|  | 100 µM | 10 µM | 1 µM |
| --- | --- | --- | --- |
| Buccalin, before | 16 | 12 | 7 |
| Buccalin, after | 16 | 7 | 10 |
| FMRFa, before | 13 | 12 | 15 |
| FMRFa, after | 11 | 8 | 15 |
| Serotonin, before | 12 | 11 | 9 |
| Serotonin, after | 13 | 8 | 11 |
| SK, before | 7 | 5 | 5 |
| SK, after | 9 | 10 | 5 |
| MilliQ, before | 15 | 18 | 10 |
| MilliQ, after | 14 | 11 | 11 |
