# Supplementary figures and images for: Analysis of rhodopsin G protein-coupled receptor orthologs reveals semiochemical peptides for parasite (Schistosoma mansoni) and host (Biomphalaria glabrata) interplay
Source: Sci Rep. 2022 May 17;12:8243. doi: 10.1038/s41598-022-11996-x (PMC9114394; doi:10.1038/s41598-022-11996-x)

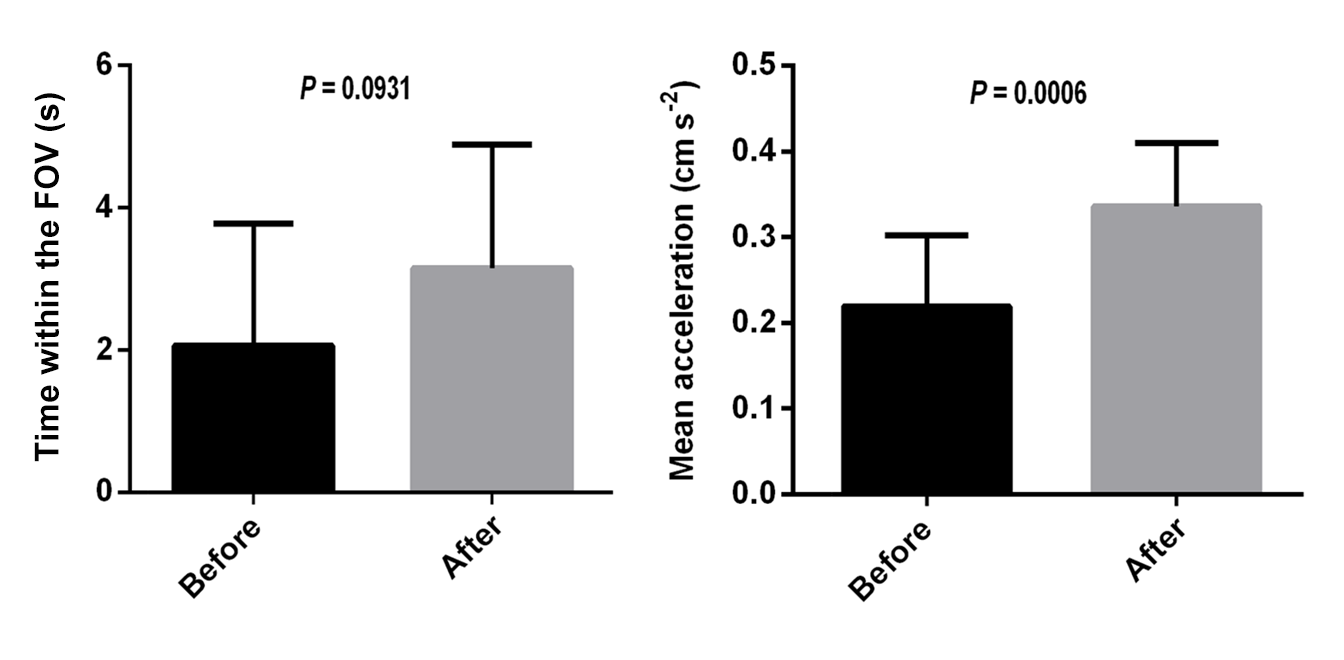

Supplement: Supplementary file 3 — Supplementary Information 3. [file 41598_2022_11996_MOESM3_ESM.tif]

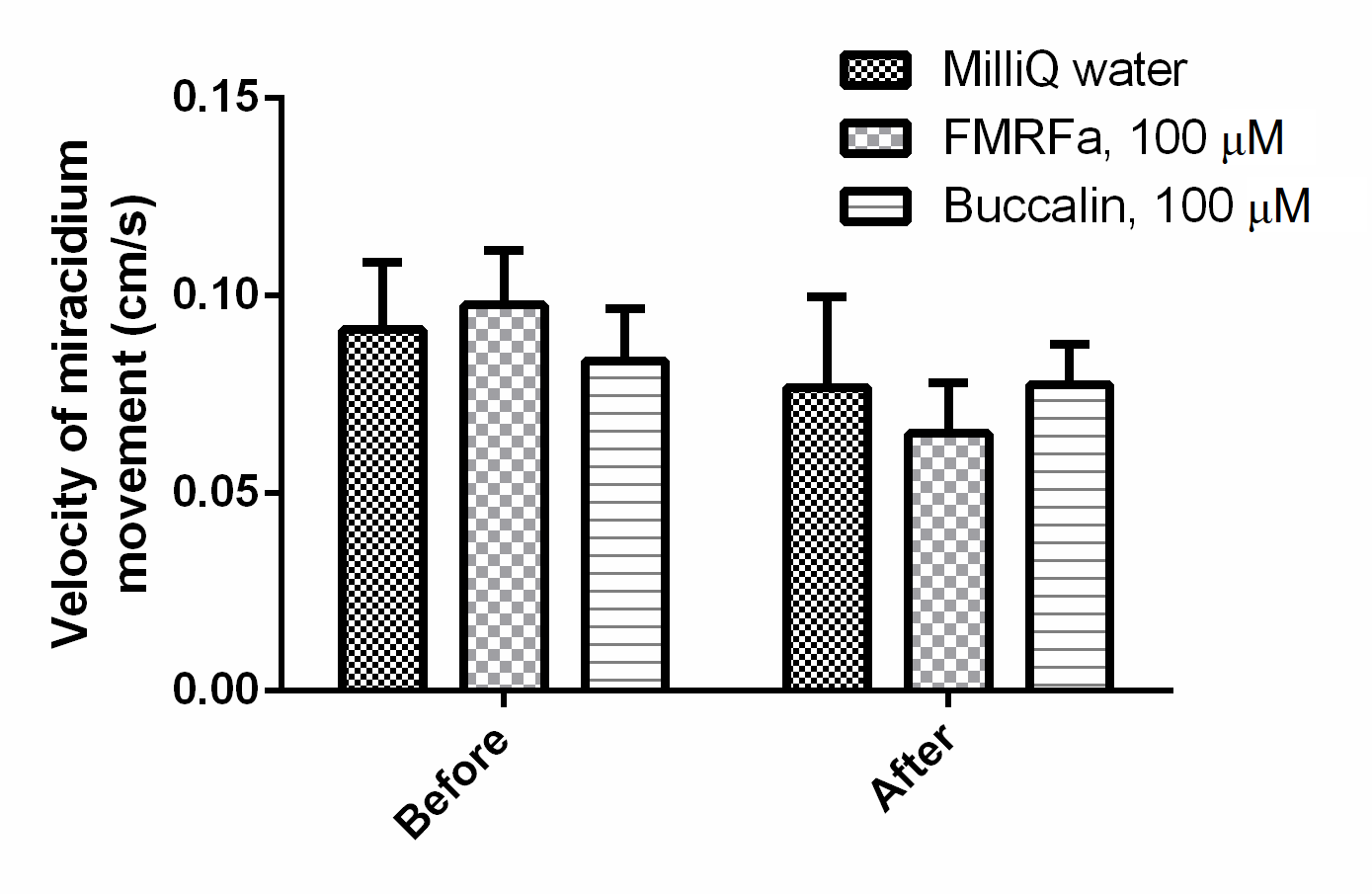

Supplement: Supplementary file 4 — Supplementary Information 4. [file 41598_2022_11996_MOESM4_ESM.tif]
