# Supplementary material for: Analysis of rhodopsin G protein-coupled receptor orthologs reveals semiochemical peptides for parasite (Schistosoma mansoni) and host (Biomphalaria glabrata) interplay
Source: Sci Rep. 2022 May 17;12:8243. doi: 10.1038/s41598-022-11996-x (PMC9114394; doi:10.1038/s41598-022-11996-x)
Supplement: Supplementary file 5 — Supplementary Information 5. [file 41598_2022_11996_MOESM5_ESM.docx]

**Additional files**

**File S1.** Numbers of the manually tracked miracidia included in behaviour analysis.

**File S2.** Comparative sequence information. (**a**) Ortholog GPCRs shared between *B. glabrata* and *S. mansoni*, including E-values and percent identity. (**b**) *B. glabrata* and *S. mansoni* miracidia GPCR protein sequences and corresponding GPCRs.

**File S3.** Graph of time duration of miracidia swimming within the recording area, and mean acceleration values, before and after the addition of 5-HT.

**File S4.** Graph showing velocity of miracidium movement before and after the addition of buccalin and FMRFa.
