# Supplementary material for: Analysis of rhodopsin G protein-coupled receptor orthologs reveals semiochemical peptides for parasite (Schistosoma mansoni) and host (Biomphalaria glabrata) interplay
Source: Sci Rep. 2022 May 17;12:8243. doi: 10.1038/s41598-022-11996-x (PMC9114394; doi:10.1038/s41598-022-11996-x)
Supplement: Supplementary file 6 — Supplementary Information 6. [file 41598_2022_11996_MOESM6_ESM.docx]

**Supplementary movies**

**Movie S1.** Movement of *S. mansoni* miracidia before and after addition of the FMRFa peptide.

**Movie S2**. Movement of *S. mansoni* miracidia before and after addition of the buccalin peptide.

**Movie S3**. Movement of *S. mansoni* miracidia before and after addition of the 5-HT.

**Movie S4**. Movement of *S. mansoni* miracidia before and after addition of the SK peptide.
